# Mechanisms of L-Triiodothyronine-Induced Inhibition of Synaptosomal Na^+^-K^+^-ATPase Activity in Young Adult Rat Brain Cerebral Cortex

**DOI:** 10.1155/2013/457953

**Published:** 2013-11-07

**Authors:** Pradip K. Sarkar, Avijit Biswas, Arun K. Ray, Joseph V. Martin

**Affiliations:** ^1^Department of Basic Sciences, Parker University, 2500 Walnut Hill Lane, Dallas, TX 75229, USA; ^2^Center for Computational & Integrative Biology, Rutgers University, 315 Penn Street, Camden, NJ 08102, USA; ^3^Department of Molecular Medicine, Bose Institute, P-1/12, CIT, Scheme VII-M, Calcutta 700054, India

## Abstract

The role of thyroid hormones (TH) in the normal functioning of adult mammalian brain is unclear. Our studies have identified synaptosomal Na^+^-K^+^-ATPase as a TH-responsive physiological parameter in adult rat cerebral cortex. L-triiodothyronine (T_3_) and L-thyroxine (T_4_) both inhibited Na^+^-K^+^-ATPase activity (but not Mg^2+^-ATPase activity) in similar dose-dependent fashions, while other metabolites of TH were less effective. Although both T_3_ and the **β**-adrenergic agonist isoproterenol inhibited Na^+^-K^+^-ATPase activity in cerebrocortical synaptosomes in similar ways, the **β**-adrenergic receptor blocker propranolol did not counteract the effect of T_3_. Instead, propranolol further inhibited Na^+^-K^+^-ATPase activity in a dose-dependent manner, suggesting that the effect of T_3_ on synaptosomal Na^+^-K^+^-ATPase activity was independent of **β**-adrenergic receptor activation. The effect of T_3_ on synaptosomal Na^+^-K^+^-ATPase activity was inhibited by the α_2_-adrenergic agonist clonidine and by glutamate. Notably, both clonidine and glutamate activate G_i_-proteins of the membrane second messenger system, suggesting a potential mechanism for the inhibition of the effects of TH. In this paper, we provide support for a nongenomic mechanism of action of TH in a neuronal membrane-related energy-linked process for signal transduction in the adult condition.

## 1. Introduction

Thyroid hormones (TH) exert major influences on the growth and development of the mammalian brain through specific nuclear receptor-mediated gene expression. Although several different isoforms of nuclear receptors for TH have been described in adult mammalian brain, their physiological function is quite unclear [1–4]. Still, adult onset of dysthyroidism develops a number of functional, neurological and psychological manifestations in humans [5–7]. In contrast to the developing brain, most of the changes resulting from hormone variations in the adult condition are reversible with the proper adjustment of circulatory TH [5–7].

Recent evidence has demonstrated that L-triiodothyronine (T_3_) is distributed, concentrated, and metabolized in the synaptosomal fraction of adult rat cerebral cortex [5, 8, 9]. Specific T_3_-binding sites have also been described in cerebrocortical synaptosomes [10, 11] and a graded binding of T_3_ to its synaptosomal receptor binding sites has been correlated with the corresponding inhibition of the Na^+^-K^+^-ATPase activities in adult rat brain [11]. TH rapidly alters *in vitro* phosphorylation of synaptosomal proteins in a dose-dependent fashion [12]. TH levels are also altered in adult rat brain in different thyroid conditions [9]. TH enhances calcium entry in adult rat brain synaptosomes [13–15], in hypothyroid mouse brain [16], and in single rat myocytes [17].

However, there is a lack of clear understanding of the mechanism(s) of action of TH in the regulation of synaptic functions in adult neurons. The present study investigates the pathways of T_3_-mediated signaling from its binding to the synaptosomal membrane receptors to the subsequent activation of second messenger system components that ultimately affect the further downstream effector molecule, the Na^+^-K^+^-ATPase. In this paper, we hypothesize a nongenomic mechanism of action of TH in neuronal membrane-related energy-linked process(es) for signal transduction in adult condition. We have used *α*- and *β*-adrenoceptor agonists and antagonists for modulation of the activity of G_s_- and G_i_-proteins of the membrane adenylate cyclase system. Portions of this work have appeared elsewhere in a preliminary form [18, 19].

## 2. Materials and Methods

### 2.1. Materials

The following compounds were purchased from Sigma Chemical Company, USA: bovine serum albumin (BSA), clonidine hydrochloride (CLO), disodium-ATP, isoproterenol hydrochloride (ISO), 2-mercaptoethanol, ouabain octahydrate, prazosin hydrochloride (PRA), phenylephrine hydrochloride (PHE), propranolol hydrochloride (PROP), sodium glutamate, 3,5,3′-L-triiodothyronine (T_3_), L-thyroxine (T_4_), 3,3′,5′-L-triiodothyronine (reverse T_3_ or r-T_3_), 3,5-L-diiodothyronine (T_2_), Tris-ATP, yohimbine hydrochloride (YOH), dibutyryl 3′,5′-cyclic adenosine monophosphate sodium salt (DB cAMP), and sodium orthovanadate.

### 2.2. Treatment of Animals

Adult male Charles Foster rats (3 months old) were housed at 25 ± 1°C in 12 h dark-12 h light conditions and fed *ad libitum* with standard rat diet and water. The animals were sacrificed by quick decapitation and the brains were removed into ice-cold 250 mM sucrose solution. The cerebral cortices were dissected out for synaptosomal fraction preparation.

### 2.3. Preparation of Synaptosomes

The synaptosomes from thecerebral cortex were prepared as described previously [20]. Briefly, the cerebral cortex was homogenized (10% weight/volume) in 0.32 M sucrose and centrifuged at 1000 g for 10 minutes to remove cell debris and nuclei. The supernatant was collected and recentrifuged at 1000 g for another 10 min. The resulting pellet was discarded and the supernatant was layered over 1.2 M sucrose and centrifuged at 34,000 g for 50 min at 4°C. The fraction collected between the 0.32 M and 1.2 M sucrose layer was diluted at 1 : 1.5 with ice-cold bidistilled water, further layered on 0.8 M sucrose, and again centrifuged at 34,000 g for 30 min. The pellet thus obtained was washed and repelleted at 20,000 g for 20 min. Synaptosomal pellets were lysed by suspending in ice-cold bidistilled water to release the occluded Na^+^-K^+^-ATPase activity.

### 2.4. Assay of Synaptosomal Na^**+**^-K^**+**^-ATPase Activity

Synaptosomal Na^+^-K^+^-ATPase activity was assayed as ouabain-sensitive ATP hydrolysis in reaction mixtures of (i) 30 mM imidazole-HCl, 130 mM NaCl, 20 mM KCl, and 4 mM MgCl_2_ and (ii) 30 mM imidazole-HCl, 4 mM MgCl_2_, and 1 mM ouabain, at pH 7.4. Both the reaction media (i) and (ii) were first preincubated *in vitro* with or without simultaneous addition of various concentrations of thyroid hormones (T_3_, T_4_) and TH-analogue (T_2_) (0.001 nM to 1 *μ*M), adrenergic drugs (1 nM for ISO, PRA, PHE and YOH; 1 nM–100 nM for CLO and PROP), glutamate (100 *μ*M), DB cAMP (1 *μ*M–5 mM), and sodium orthovanadate (10 nM–2 mM) followed by addition of the synaptosomal lysates, each containing 20–50 *μ*g synaptosomal protein, at 0°C for 60 minutes in dark. To get a steady-state ouabain binding, both the assay media (i) and (ii), with and without ouabain, respectively, as described above, were preincubated for 60 min at 0°C in the dark, followed by a 5-min incubation at 37°C to equilibrate the temperature. The reaction was started by adding 4 mM Tris-ATP and incubated at 37°C for 10 min. An aliquot of 100 *μ*L of 10% sodium dodecylsulfate was added to stop the enzymatic reaction. The inorganic phosphate (P_i  _) formed was determined in the reaction mixture [21]. Na^+^-K^+^-ATPase activity was calculated as difference in the P_i_ content between media (i) and (ii) and expressed as *μ*mols P_i_/h/mg protein [22]. The ouabain-sensitive portion of the total ATPase (Na^+^-K^+^-Mg^2+^-ATPase) was determined from the P_i_ released in the medium (i) minus that in medium (ii). The P_i_ released from the reaction medium (ii) was used for determination of the synaptosomal Mg^2+^-ATPase activity. Synaptosomal Mg^2+^-ATPase activity, therefore, was assayed as the ouabain-insensitive ATP hydrolysis.

### 2.5. Measurement of Protein

Synaptosomal protein content was measured using bovine serum albumin as a standard [23].

### 2.6. Statistical Analysis

Results are expressed as the mean ± SEM of 3-4 separate experiments or as mentioned. Each experiment was made from six rats. The statistical analysis of the data was performed by Student's *t*-test, considering *P* < 0.05 as the significance level. The data for multiple groups were also analyzed by one-way ANOVA followed by Student Newman-Keuls post-hoc comparisons using Sigmastat software. Nonlinear regression analysis was performed using GraphPad Prism software.

## 3. Results

### 3.1. Effects of T_**3**_ and Metabolites on Na^**+**^-K^**+**^-ATPase Activity

**Figure 1:**
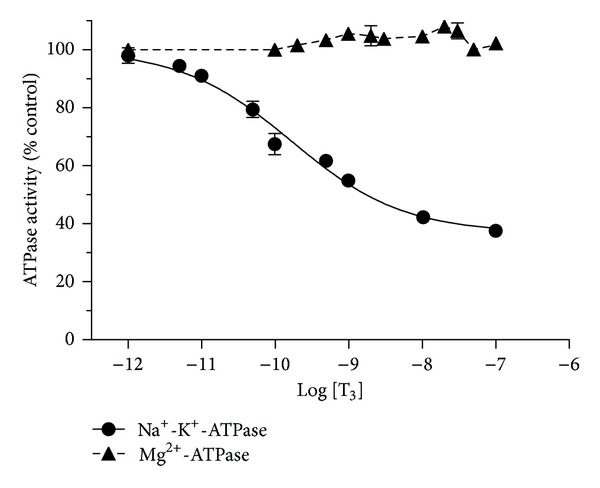
Inhibitory effect of various doses (0.001 nM–100 nM) of T_3_ on synaptosomal Na^+^-K^+^-ATPase or Mg^2+^-ATPase activity, *in vitro*. The data are represented as mean ± SEM of ten separate experiments, taking six animals in each group. The vertical lines denote SEM. Filled circles indicate Na^+^-K^+^-ATPase while filled triangles indicate Mg^2+^-ATPase activity.

**Figure 2:**
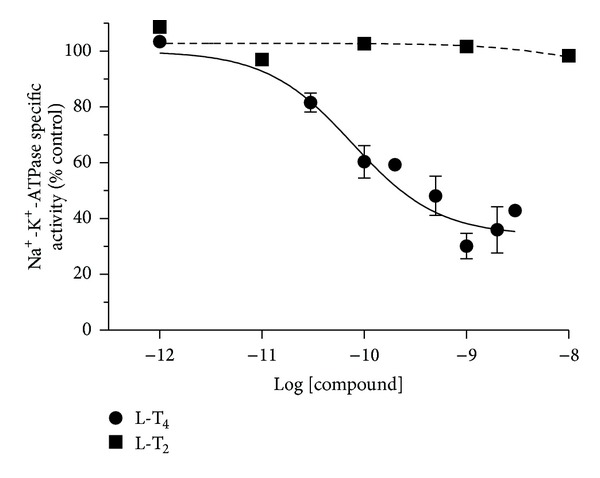
Inhibitory effect of various doses (0.001 nM–10 nM) of T_4_ or T_2_ on synaptosomal Na^+^-K^+^-ATPase activity, *in vitro*. The data are represented as mean ± SEM of four separate experiments, taking six animals in each group. The vertical lines denote SEM. Filled circles indicate effects of T_4_ on Na^+^-K^+^-ATPase activity while filled squares indicate effects of T_2_.

*In vitro* addition of various doses of T_3_ to the synaptosomal fraction (which is devoid of cell nuclei) confirmed our previous observation [11] and showed nearly the same trend of a dose-dependent inhibition (IC_50_ = 166.4 ± 55.0 pM; maximal inhibition = 63.2 ± 3.4% at 95% confidence levels) of Na^+^-K^+^-ATPase activity. No significant effect of T_3_ was noticed on the Mg^2+^-ATPase specific activity (Figure 1). T_4_ had a similar inhibitory effect as T_3_ on Na^+^-K^+^-ATPase activity (IC_50_ = 77.2 ± 31.8 pM; maximal inhibition = 66.5 ± 7.2%), while T_2_ had minimal effects (Figure 2). Furthermore, the same range of doses (10^−12^–10^−8^ M) of r-T_3_ did not inhibit either Na^+^-K^+^-ATPase or Mg^+2^-ATPase activities (data not shown).

### 3.2. Effect of T_**3**_ and *β*-Adrenergic Agonists/Antagonists on Na^**+**^-K^**+**^-ATPase Activity

**Figure 3:**
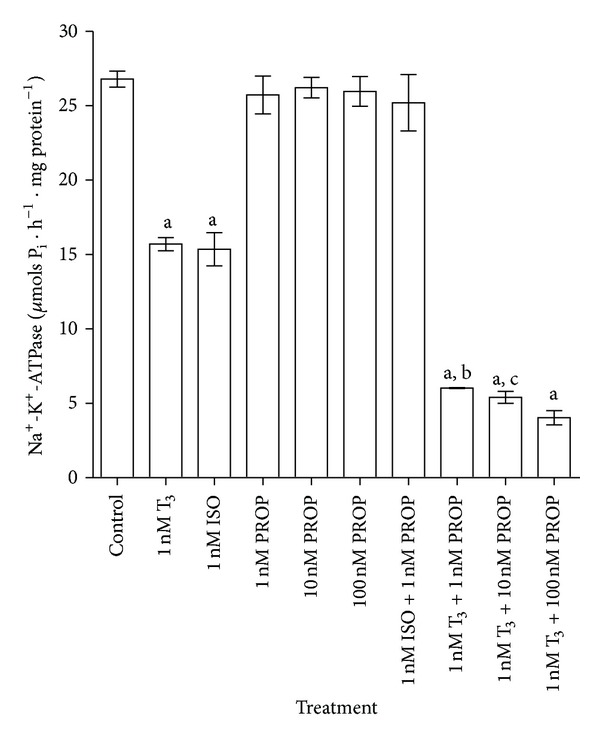
Effect of T_3_ on synaptosomal Na^+^-K^+^-ATPase activity and its modulation by a *β*-adrenergic receptor agonist (ISO) and a *β*-adrenergic receptor antagonist (PROP) *in vitro*. A half-maximally effective dose of T_3_ (1 nM) was chosen from the dose-response curve for T_3_ in Figure 1. The data are represented as mean ± SEM of five separate experiments taking six animals in each group. ^a^ *P* < 0.001, compared to the control group. ^b^ *P* < 0.001 and ^c^ *P* < 0.05, compared to T_3_ (1 nM) + PROP (100 nM) group (one-way ANOVA followed by Newman-Keuls test). The vertical lines denote SEM.

Equimolar doses (1 nM) of T_3_ and the nonselective *β*-adrenergic agonist ISO were added separately *in vitro*, inhibited the Na^+^-K^+^-ATPase enzyme activity by 41.3% and 42.6%, respectively (Figure 3). The nonselective *β*-adrenergic antagonist PROP alone did not alter the enzyme activity at different doses (1 nM, 10 nM, and 100 nM). The inhibitory action of ISO (1 nM) on the Na^+^-K^+^-ATPase activity was counteracted by PROP (1 nM), whereas PROP could not block T_3_-mediated inhibition of the enzyme activity. Instead PROP potentiated the T_3_-mediated inhibition of the enzyme activity in a dose-dependent manner. Significant differences in the potentiation of the T_3_ effect (1 nM) by PROP on Na^+^-K^+^-ATPase activity were noticed between 1 nM and 100 nM (*P* < 0.001) and between 10 nM and 100 nM (*P* < 0.05) doses (Figure 3).

### 3.3. Effects of T_**3**_ and *α*-Adrenergic Agonists/Antagonists on Na^**+**^-K^**+**^-ATPase Activity

**Figure 4:**
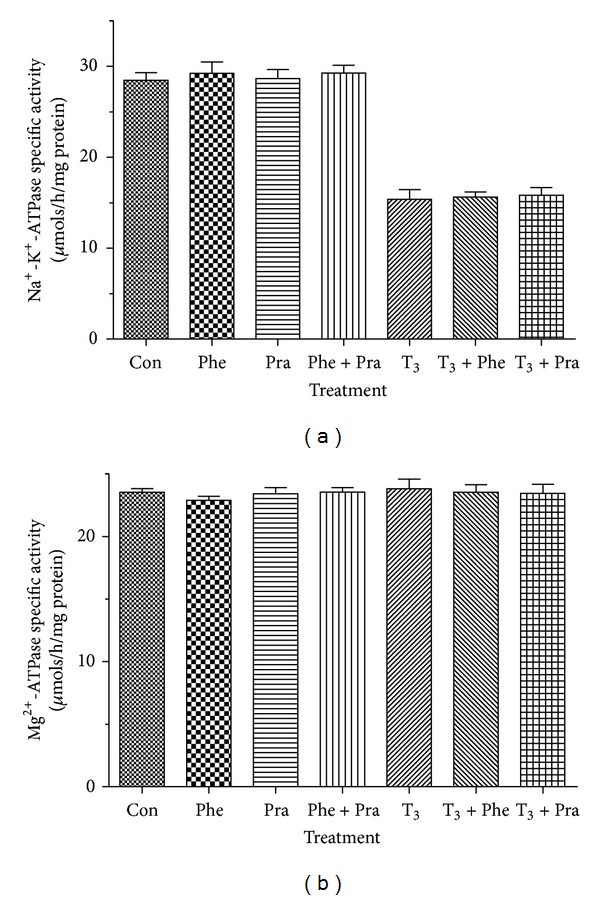
Modulation of the T_3_ action on synaptosomal Na^+^-K^+^-ATPase activity by a selective *α*_1_-adrenergic agonist (PHE) and a selective *α*_1_-antagonist (PRA) *in vitro*. A half-maximally effective dose of T_3_ (1 nM) was chosen from the dose-response curve for T_3_ in Figure 1. The doses for PHE and PRA used for the *in vitro* experiment were 1 nM in each case. The data are represented as mean ± SEM of five separate experiments, taking six animals in each group. The vertical lines denote SEM.

The effects of *in vitro* addition of 1 nM doses of PHE (selective *α*_1_-adrenergic receptor agonist) and PRA (*α*_1_-adrenergic receptor antagonist) on synaptosomal Na^+^-K^+^-ATPase activity or Mg^2+^-ATPase activity were minimal (Figure 4). Furthermore, 1 nM doses of PHE or PRA did not alter the inhibitory effect of 1 nM T_3_ on Na^+^-K^+^-ATPase activity, nor did it change the Mg^2+^-ATPase activity, *in vitro* (Figure 4).

**Figure 5:**
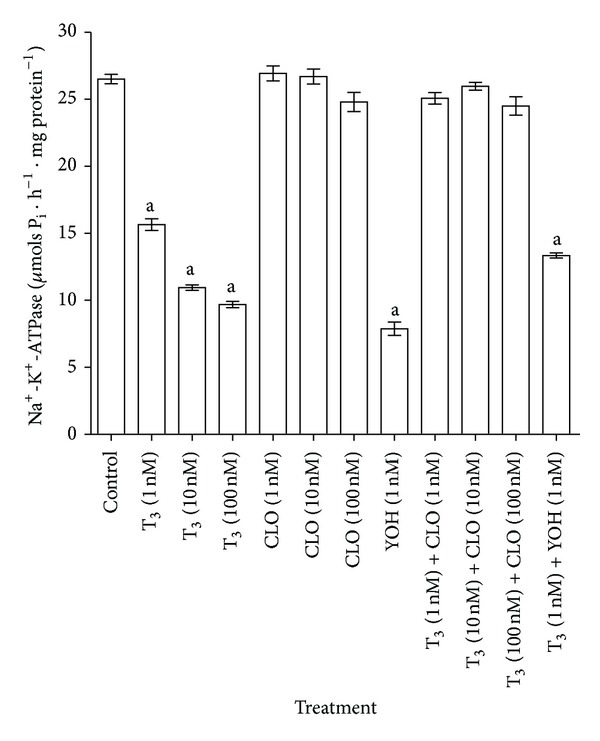
Modulation of the T_3_ action on synaptosomal Na^+^-K^+^-ATPase activity by an *α*_2_-adrenergic agonist (CLO) and an *α*_2_-adrenergic antagonist (YOH) *in vitro*. The data are represented as mean ± SEM of six separate experiments taking six animals in each group. ^a^ *P* < 0.001, compared to the control group (one-way ANOVA followed by Newman-Keuls test). The vertical lines denote SEM.

Similarly, *in vitro* addition of CLO (*α*_2_-adrenergic agonist) at different doses did not elicit significant changes in the synaptosomal Na^+^-K^+^-ATPase activity (Figure 5). However, when CLO was added in the presence of an equimolar dose of T_3_, the inhibitory effect of T_3_ on the Na^+^-K^+^-ATPase activity was completely counteracted. The effect of T_3_ on the enzyme activity remained prominent at a 100 nM dose of T_3_ (100 nM T_3_: 10.29 ± 0.2 *μ*mols P_i_/h/mg protein; Control: 26.22 ± 0.2 *μ*mols P_i_/h/mg protein) along with 1 nM CLO (100 nM T_3_ + 1 nM CLO: 15.23 ± 0.4 *μ*mols P_i_/h/mg protein); however, 1 nM CLO attenuated the effect of T_3_ (100 nM) by 32% more towards the control value (data not shown graphically). The *α*_2_-adrenergic receptor antagonist YOH also inhibited synaptosomal Na^+^-K^+^-ATPase activity (Figure 5). Inhibition of the enzyme activity in the presence of both 1 nM T_3_ and 1 nM YOH was found to be intermediate between the levels of inhibition by either compound alone, although there were no significant differences between these groups (Figure 5).

### 3.4. Effect of T_**3**_ and Glutamate on Na^**+**^-K^**+**^-ATPase Activity

**Figure 6:**
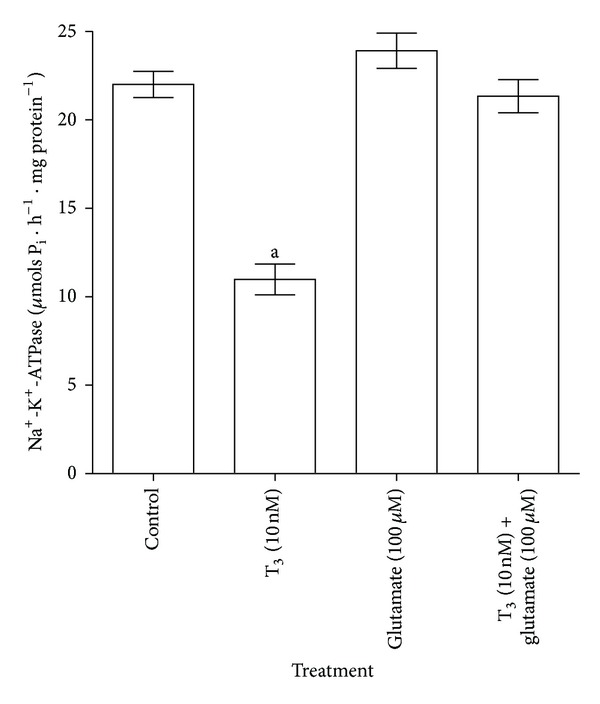
Modulatory effect of glutamate on T_3_-induced inhibition of synaptosomal Na^+^-K^+^-ATPase activity in cerebral cortex, *in vitro*. A higher dose of T_3_ (10 nM) was chosen from the T_3_ dose-response curve, considering its greater inhibitory action on the Na^+^-K^+^-ATPase activity and to observe the effect of 100 *μ*M glutamate on this T_3_-induced inhibition. The data are represented as mean ± SEM of four separate experiments taking six animals in each group. ^a^ *P* < 0.001, compared to the control group (one-way ANOVA followed by Newman-Keuls test).

*In vitro* addition of 100 *μ*M glutamate alone did not alter the synaptosomal Na^+^-K^+^-ATPase activity compared to control values, whereas, addition of 100 *μ*M glutamate showed complete attenuation of T_3_ (10 nM)-mediated inhibition of synaptosomal Na^+^-K^+^-ATPase activity in adult rat cerebral cortex (Figure 6). A higher dose of T_3_ (10 nM) was chosen, in order to test the effect of glutamate against a greater inhibitory action on the Na^+^-K^+^-ATPase activity.

### 3.5. Effect of DB cAMP and T_**3**_ on Na^**+**^-K^**+**^-ATPase Activity

**Figure 7:**
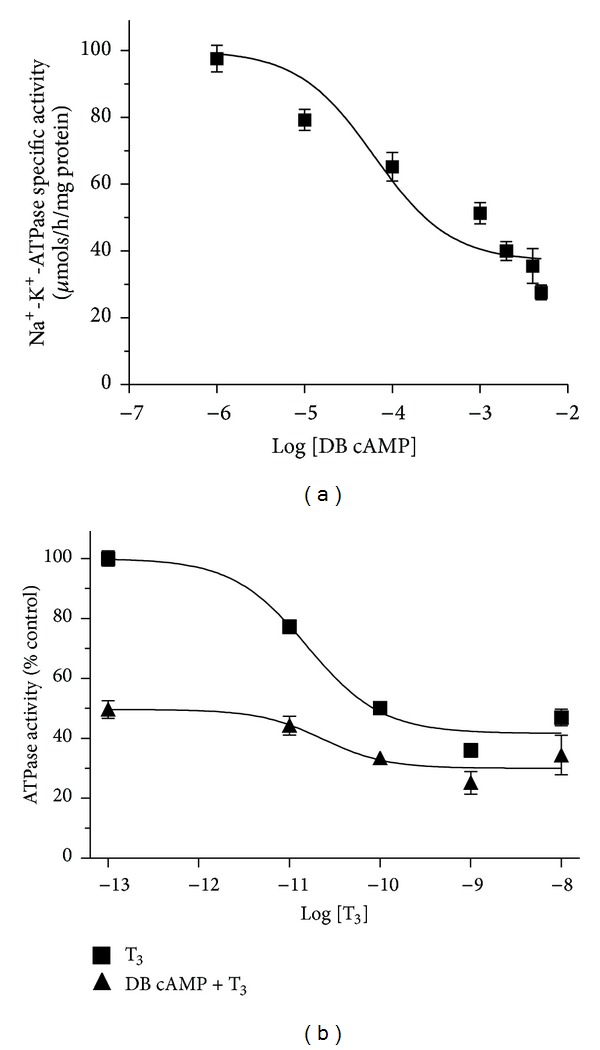
Influence of DB cAMP and T_3_ on synaptosomal Na^+^-K^+^-ATPase activity, *in vitro*. (a) Inhibitory effect of various doses of DB cAMP on synaptosomal Na^+^-K^+^-ATPase activity, *in vitro*. The data are represented as mean ± SEM of four separate experiments, taking six animals in each group. The vertical bars denote SEM. (b) Interaction of the effects of of DB cAMP and T_3_ on synaptosomal Na^+^-K^+^-ATPase activity, *in vitro*. Filled squares indicate effects of graded doses of T_3_ (0.001 nM–10 nM) alone on Na^+^-K^+^-ATPase activity while filled triangles indicate effects of the 0.2 mM dose of DB cAMP with graded doses of T_3_ (0.1 pM–1 *μ*M).

To study the effect of DB cAMP on modulation of Na^+^-K^+^-ATPase activity by T_3_, first a dose response experiment with various concentrations of DB cAMP (0.001 mM to 5 mM) was performed. *In vitro* addition of DB cAMP showed a typical sigmoidal curve with gradual decrease in the Na^+^-K^+^-ATPase activity to a maximal inhibition at 0.2 mM (Figure 7(a)). From this standardization, we chose to use a 0.2 mM final concentration of DB cAMP for further experiments. *In vitro* addition of DB cAMP (0.2 mM) with and without various doses of T_3_ (0.001 nM–10 nM) was examined for effects on Na^+^-K^+^-ATPase activity (Figure 7(b)). T_3_-induced inhibition of synaptosomal Na^+^-K^+^-ATPase activity was further depressed in the presence of 0.2 mM DB cAMP. However, the two curves appeared to converge at the highest doses of T_3_.

### 3.6. Influence of Sodium Orthovanadate on Modulation of Na^**+**^-K^**+**^-ATPase Activity by T_**3**_

**Figure 8:**
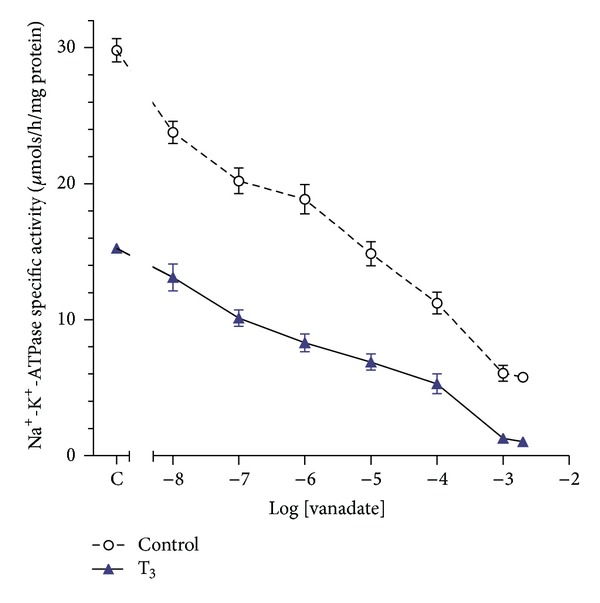
Modulation of the T_3_ action on synaptosomal Na^+^-K^+^-ATPase activity by sodium orthovanadate. A higher dose of T_3_ (10 nM) was chosen from the T_3_ dose-response curve considering its greater inhibitory action on the Na^+^-K^+^-ATPase activity and to observe the effect of graded doses (1 nM–2 mM) of sodium orthovanadate on this T_3_-induced inhibition. The data are represented as mean ± SEM of four separate experiments, taking six animals in each group. The vertical bars denote SEM. Open circles indicate mean values for a set of control incubations without T_3_. Filled triangles indicate the results of a set of incubations with 10 nM T_3_.

The *in vitro* effect of sodium orthovanadate, a protein tyrosine phosphatase inhibitor, was examined in cerebrocortical synaptosomes. The cerebrocortical synaptosomes were treated with a fixed dose of T_3_ (10 nM) with or without different doses of sodium *o*-vanadate (Figure 8). A higher dose of T_3_ (10 nM) was chosen from the T_3_ dose-response curve, considering its greater inhibitory action on the Na^+^-K^+^-ATPase activity. T_3_ caused an inhibition of Na^+^-K^+^-ATPase specific activity, and this effect was enhanced by sodium orthovanadate in a dose-dependent way. In general, the effects of sodium orthovanadate and T_3_ appeared to be additive until the Na^+^-K^+^-ATPase specific activity was completely inhibited.

## 4. Discussion

The objective of the present investigation was to search for possible mechanisms for the inhibition by TH of synaptosomal Na^+^-K^+^-ATPase activity in adult rat cerebral cortex.

Initial studies examined the specificity of the effect according to the pattern of iodination of the hormone derivatives (Figures 1 and 2). *In vitro* inhibitory effect of T_3_ on synaptosomal Na^+^-K^+^-ATPase activity supported our previous observation and showed nearly the same trend of a dose-dependent inhibition of Na^+^-K^+^-ATPase activity [11]. In addition to our earlier report, the current study showed an insignificant effect of T_3_ on the synaptosomal Mg^2+^-ATPase specific activity (Figure 1). *In vitro* addition of T_4_ also indicated similar pattern of inhibitory influence on the synaptosomal Na^+^-K^+^-ATPase activity, like the effect of T_3_, with no significant changes on the Mg^2+^-ATPase activity. The effects of TH on Na^+^-K^+^-ATPase activity seemed to be specific for compounds with 2 iodine atoms on the inner ring, as T_2_ and r-T_3_ were without activity in the current studies. T_3_ was less potent than T_4_. It is consistent with reports of the relative affinities of the two compounds for a cell surface receptor, integrin *α*_v_ *β*_3_ known to mediate a variety of nongenomic effects of THs [24].

Binding of T_4_ to integrin *α*_v_ *β*_3_ causes internalization of the receptor and nongenomically promotes phosphorylation of mitogen-activated protein kinase/extracellular regulated kinase 1 and 2 (MAPK/ERK_1/2_) in the CV-1 line of monkey fibroblasts [24]. A similar mechanism seems likely in chick chorioallantoic membrane [25]. Following the internalization of the integrin *α*_v_ *β*_3_, the *α*_v_ monomer is translocated to the nucleus, where it may transcriptionally regulate expression of protein [26]. TH causes lungs to rapidly (within hours) increase alveolar fluid clearance [27] and to express increased Na^+^-K^+^-ATPase protein by a MAPK/ERK_1/2_-dependent pathway [28]. Note, however, that the current finding of an immediate effect to decrease Na^+^-K^+^-ATPase activity could not be due to a mechanism involving transcriptional regulation, since the synaptosomal preparation is devoid of cell nuclei. It is also suggested that some of the effects of T_3_ stimulation of the integrin *α*_v_ *β*_3_ could be more direct than the nuclear interaction [29].

A potential mechanism for the inhibitory effects of TH in the present study might be the regulation of phosphorylation of Na^+^-K^+^-ATPase or a modulatory molecule. It is well known that catecholamine-mediated phosphorylation of Na^+^-K^+^-ATPase inhibits enzymatic activity in Chinese hamster ovary (CHO) cells, but not through a process of internalization of the enzyme [30–32]. Intriguingly in this respect, one of the proteins found to be phosphorylated at the tyrosyl residue in synaptosomes treated for 5 s with TH had a molecular weight of 95 kD [12], matching the size of *α*-subunit of Na^+^-K^+^-ATPase [33].

The significant inhibition of the synaptosomal Na^+^-K^+^-ATPase activity *in vitro* by T_3_ confirmed our previous *in vivo* observations [22]. In order to characterize this inhibitory influence of THs on the synaptosomal membrane, we intended to study the effect of adrenergic receptor agonists and antagonists which regulate guanine nucleotide binding proteins (G-proteins) via their activating and inhibitory actions. Both T_3_ and ISO (*β*-adrenergic receptor agonist) showed an analogous but independent (parallel) inhibitory effect on the enzyme activity (Figure 3). ISO-induced inhibition of Na^+^-K^+^-ATPase activity was blocked by PROP (*β*-adrenergic receptor blocker) indicating that the synaptosomal membrane interaction with ISO was likely a *β*-adrenoceptor-mediated event, potentially coupled to G_s_-protein. However, PROP was completely unable to block T_3_-mediated inhibition of synaptosomal Na^+^-K^+^-ATPase activity. This clearly indicated that T_3_-mediated inhibition of the enzyme activity was not coupled to *β*-adrenoceptor, but rather, may have had a similar effect through another kind of receptor. The augmentation of the T_3_ effect by PROP appeared to be a type of synergistic action, the mechanism of which remains unclear at present. Increased activity of adenylate cyclase caused by THs, independent of propranolol blockade, has been shown in cultured cerebral cells from embryonic mice, suggesting that the effect of T_3_ was not mediated through a *β*-adrenergic-dependent system [34]. The T_3_-induced increase in sodium current in neonatal rat myocytes also could not be blocked by PROP, whereas it was antagonized by amiodarone, a nonspecific blocker of *β*-adrenoceptor, suggesting that the effects were not mediated through *β*-adrenergic signaling pathways [35]. However, *β*-adrenoceptor blockade by chronic subcutaneous delivery of PROP for 14 days has been shown to downregulate levels of TH receptor TR *α*_1_-mRNA and *β*_1_-mRNA in mouse heart, which may influence the genomic effect of the hormone [36].

Next, we wanted to check for the role of an *α*_1_-adrenergic receptor agonist and antagonist. Agonists for the *α*_1_-adrenergic receptor mediate their actions through G_q_ protein followed by activation of phospholipase C and subsequent production of the second messengers inositol triphosphate and diacylglycerol, an activator of protein kinase C [37]. Neither PHE (selective *α*_1_ agonist) nor PRA (*α*_1_ antagonist) had an influence on Na^+^-K^+^-ATPase activity. Furthermore, neither compound interacted with the effects of T_3_. Mg^2+^-ATPase activity remained unaltered when treated with either of these *α*_1_-adrenergic drugs (agonist and antagonist) and T_3_, alone or in combination (Figure 4). These results suggest that the effects of T_3_ on Na^+^-K^+^-ATPase activity do not share common mechanisms with *α*_1_-receptors.

On the other hand, CLO, an *α*_2_-adrenergic receptor agonist (Figure 5), and glutamate (Figure 6), possibly acting via a metabotropic glutamate receptor (mGluR), blocked T_3_-induced inhibition of Na^+^-K^+^-ATPase activity. Neither CLO nor glutamate showed any significant effect on the Na^+^-K^+^-ATPase activity in rat hippocampus and frontal cortex homogenates [38]. One possibility would be that the counteraction of the effect of T_3_ on synaptosomal Na^+^-K^+^-ATPase by CLO and glutamate might be mediated through the inhibition of adenylate cyclase activity with the activation of inhibitory G-protein (G_i_) followed by the inhibition of cAMP synthesis and the protein phosphorylation cascade mechanism. It is well known that *α*_2_-adrenergic agonists act through stimulation of G_i_-protein [18, 19, 39, 40].

Association of the glutamate transporter with Na^+^-K^+^-ATPase in synaptosomes has been implicated by their correlated regulation via protein kinases [41]. Glutamate also has been reported to inhibit adenylate cyclase activity in rat hippocampal synaptosomes [39, 40, 42, 43], as well as in striatal and cerebrocortical neurons, both in intact cells and membranes [40] via metabotropic glutamate receptors (mGluRs), which are coupled to effector systems through GTP binding proteins. In fact, in the nucleus tractus solitarius of adult brain, it was shown that an antibody to the G_i_ inhibited the effects of mGluRs [44]. mGluR_1_ and mGluR_5_ subtypes are coupled to phosphatidyl inositol hydrolysis/Ca^2+^-signal transduction. mGluR_1_ has also been shown to stimulate release of arachidonic acid and to increase cAMP formation. The mGluR_2_, mGluR_3_, mGluR_4_, and mGluR_5_ subtypes appear to be coupled to inhibition of cAMP synthesis, but differ in their agonist selectivity. mGluR_2_ and mGluR_3_ mRNAs are highly expressed in the cerebral cortex [40, 42, 43]. Activation of mGluR has been shown to counteract *β*-adrenoceptor-mediated inhibition of afterhyperpolarization in hippocampal neurons of the CA1 area. This has been suggested to be by mGluR-mediated activation of protein kinase C, which inhibited adenylate cyclase pathways [42, 43]. The physiological functions of these mGluRs are still being clarified. Thus, T_3_ action in adult rat synaptosomal membrane, ultimately to inhibit the effector molecule Na^+^-K^+^-ATPase, might be mediated through G_s_ stimulation. mGlu receptors may then have some regulatory roles in counteracting T_3_-induced action.

Our observation showed that DB cAMP (a nonhydrolyzable form of cAMP and activator of cAMP-dependent protein kinases) had a T_3_-like effect on Na^+^-K^+^-ATPase activity (Figure 7). Furthermore, the *in vitro* addition of increased doses of T_3_ lowered the slope of the dose-response curve for DB cAMP. Such a finding might be consistent with a related mechanism for the effects of DB cAMP and T_3_, and would not represent merely additive effects of two distinct mechanisms. Our previous observations suggested that the phosphorylation status of certain synaptosomal proteins could be mediated via cAMP- and/or Ca^2+^-dependent pathways [12, 45]. A differential stoichiometry of phosphorylation of the *α*-subunit of the Na^+^-K^+^-ATPase by protein kinase A and protein kinase C has been shown to inhibit this enzymatic activity in shark rectal gland, rat renal cortex, and basolateral membrane vesicles from rat renal cortex [46].

The effect of the protein tyrosine phosphatase inhibitor sodium orthovanadate [47] appeared to be additive to the effect of T_3_, implying that there could be a separate mechanism of action of the two compounds (Figure 8). Since vanadate is a blocker of tyrosine phosphatase activity, it also could be speculated that T_3_-induced inhibition of Na^+^-K^+^-ATPase activity is further suppressed by synergistic action by vanadate via keeping the enzyme in its phosphorylated form, causing inhibition of its activity. A point to note here is that the *α*-subunit is the catalytic subunit and its phosphorylation causes inhibition of this enzyme [46]. T_3_ appears not to have the inhibitory effect on Na^+^-K^+^-ATPase activity by an influence on phosphatase activity.

## 5. Conclusion

Our results regarding T_3_ action in relation to the inhibition of synaptosomal Na^+^-K^+^-ATPase are consistent with a T_3_-synaptosomal membrane component binding site interaction sensitive to the activation of G_i_-protein. Such a membrane binding component might interact with a G_s_-protein, resulting in increased synthesis of cAMP. The membrane Na^+^-K^+^-ATPase is involved in several aspects of physiological processes. In the neuron, its inhibition is linked with neurotransmitter release [46]. Hence, the present study provides further evidence of a nongenomic membrane-related action of T_3_ in the mature mammalian synaptosome. Understanding of the mechanism of action of TH in adult mammalian brain has major implications in the higher mental functions and in the regulation of several neuropsychiatric disorders developed in thyroid dysfunctions in adult humans.
